# Replacing Standard Reporters from Molecular Cloning Plasmids with Chromoproteins for Positive Clone Selection

**DOI:** 10.3390/molecules23061328

**Published:** 2018-05-31

**Authors:** Margarita Daniela Tafoya-Ramírez, Felipe Padilla-Vaca, Ana Patricia Ramírez-Saldaña, Josué Daniel Mora-Garduño, Ángeles Rangel-Serrano, Naurú Idalia Vargas-Maya, Luz Janeth Herrera-Gutiérrez, Bernardo Franco

**Affiliations:** Departamento de Biología, Universidad de Guanajuato, Noria Alta, 36050 Guanajuato, Mexico; max_tafas@hotmail.com (M.D.T.-R.); padillaf@ugto.mx (F.P.-V.); anapatricia.rs.18@gmail.com (A.P.R.-S.); jhosdj@live.com.mx (J.D.M.-G.); arangel@ugto.mx (Á.R.-S.); naudalia@hotmail.com (N.I.V.-M.); luz.janeth.herrera@hotmail.com (L.J.H.-G.)

**Keywords:** molecular cloning, plasmid, clone selection, AmilCP chromoprotein, protein expression

## Abstract

Cloning and expression plasmids are the workhorses of modern molecular biology. Despite the pathway paved by synthetic biology, laboratories around the globe still relay on standard cloning techniques using plasmids with reporter proteins for positive clone selection, such as β-galactosidase alpha peptide complementation for blue/white screening or *ccdB*, which encodes for a toxic DNA gyrase. These reporters, when interrupted, serve as a positive clone detection system. In the present report, we show that molecular cloning plasmids bearing the coding sequence for a 25.4 kDa protein, AmilCP, encoded by a 685 bp gene, that is well expressed in *Escherichia coli*, render blue-purple colonies. Using this reporter protein, we developed and tested a cloning system based on the constitutive expression of the non-toxic AmilCP protein, that once interrupted, the loss of purple color serves to facilitate positive clone selection. The main advantage of this system is that is less expensive than other systems since media do not contain chromogenic markers such as X-gal, which is both expensive and cumbersome to prepare and use, or inductors such as IPTG. We also designed an inducible expression plasmid suitable for recombinant protein expression that also contains AmilCP cloning selection marker, a feature not commonly found in protein expression plasmids. The use of chromogenic reporters opens an important avenue for its application in other organisms besides *E. coli* for clone selection or even for mutant selection.

## 1. Introduction

Plasmids have been used for several decades now as vehicles for cloning and sequencing, mutagenesis protocols, protein engineering and expression, and shuttles between organisms among many others [1]. Since the generation of the first recombinant molecule by Jackson and colleagues [2], giant steps towards the comprehension of living organism’s fundamental processes have been made and the birth of biotechnology was made possible [3]. The more diverse and complex the plasmids are, the more applications and broader hosts we can genetically engineer. To expand our knowledge regarding gene function in specific organisms, plasmids are the most important tool for gene silencing experiments, protein overexpression and more recently, the generation of genome editing tools, rendering plasmids as the prime choice for modern biology research [3,4]. Still, the biology of these diverse molecules remains surprising for researchers due to their ecological role and the evolutionary pressure for the host [5].

We have acquired reasonably enough information to understand the works behind plasmid biology and their usefulness, perhaps the strongest tool for novel plasmid development can be found in synthetic biology, powered by specific software and the ever-reducing costs in DNA sequencing and synthesis [4]. Even with all the advances thus far reported in the literature, there are some aspects of cloning plasmids that can be improved. For instance, the selection of positive clones.

Around the world, many labs still relay on plasmids bearing a selective marker for successful clone selection, such as blue/white screening, inactivation of toxic genes, GFP or fluorescent protein expressing vectors wherein upon cloning the fluorescence is absent, other reporter genes such as phosphatases or biosynthetic genes that produces color when uninterrupted by a cloning fragment [1,6]. These systems are restricted depending on the bacterial host where molecular cloning is desired due to a limitation in codon usage and regulatory elements that drive reporter expression.

The most common system in cloning plasmids (for primary cloning experiments such as genomic or cDNA libraries, sequencing, site-directed mutagenesis among others) is the α-fragment (amino-terminal fragment) interruption of the β-galactosidase-encoding gene (*lacZα*) [7,8], a phenomenon that has been characterized to the molecular level, showing the distinctive nature of the β-galactosidase enzyme [9]. This kind or reporters have an implicit high expense in cloning procedures, associated with the need of the use of 5-bromo-4-chloro-3-indolyl-β-d-galacto-pyranoside (X-gal) when selecting β-galactosidase inactivation and IPTG induction of the reporter enzyme [10,11]. This substrate cannot be stored for long periods of time and it is light sensitive. Some success can be determined without the indicator X-gal by colony size, but in our labs, this observation has been proven unsuccessful many times [12]. Also, long exposure to X-gal (in DMF as solvent) leads to colony death, making it difficult to recover colonies after some time [13]. Plasmids depending on α-fragment interruption have other alternatives such as the S-gal substrate [14], which is autoclavable and microwaveable but expensive (1 g costs $663.00 USD versus $740.00 USD for 2.5 g of X-gal, purchased from Sigma-Aldrich (St. Louis, MO, USA). Alternatively, mutants in *galE* gene that encodes for the galactose epimerase are sensitive to galactose due to the accumulation of the toxic intermediate UDP-galactose. In *galE* deficient backgrounds, in the presence of lactose (with a lower cost compared to X-gal, $50.50 USD per kilogram, Sigma-Aldrich), survive if the *lacZ* complementation does not occur [15]. The only two relevant setbacks are observed in this system, one is that the recipient strains should bare a *galE* mutation and secondly, the cloning plasmids should exhibit high β-galactosidase activity, which in turn is also induced by IPTG.

Other efforts still implicate the use of chromogenic derivatives for positive clone selection, for example in plasmids for *Streptomyces* where the inactivation of the indigionin synthetase (*idgS*) renders a blue/white detection system to facilitate the generation of gene knockouts [16]. Alternatively, the use of melanin-synthesizing gene generated plasmid pIJ702, which is functional and efficient in *Streptomyces* strains lacking additional melanin-synthesizing genes [17]. This system is impaired for its use in *E. coli* due to the high G+C content of the genus *Stremptomyces* genomes. Nevertheless, these are alternatives to the standard *lacZα* clone selection methods.

Another extensively used reporter is the enzyme β-glucuronidase (*udiA* gene), for both plasmid cloning or reporter assays in several organisms including plants and is less expensive than β-galactosidase assays [18]. The only drawback of this system is the *in vivo* selection is more complicated, due to the requirement of 4-methylumbelliferyl-β-d-glucuronide and UV light screening [19].

An interesting method relies on the use of uroporphyrinogen III methyltransferase (*cob*A or *cys*G genes) in plasmids, generating an accumulation of porphyrinoid compounds. When cells harboring intact *cob*A or *cys*G genes, when cells are illuminated with ultraviolet light, the cells fluoresce with a bright red color [20]. Cloning in these open reading frames interrupts their expression and colonies are non-fluorescent. Again, this method exposes cells to UV light which can generate unwanted mutations.

Many vectors rely on the interruption of toxic genes, the most widespread is the *ccd*B gene, which encodes for a toxic DNA topoisomerase and currently, the Zero Background™ system from Invitrogen (Carlsband, CA, USA) is the most used system [21]. Although, this method relies on strains resistant to CcdB to generate enough plasmid for cloning, for example, One Shot^®^ *ccdB* Survival™ 2 T1R cells (Thermo Fisher Scientific, Waltham, MA, USA).

The selection of positive clones is still applied for cloning and sequencing, with cheaper procedures this technique can be applied even in diagnostics. One exceptional example is in the diagnosis of genetic mutations, such as described by Zhang [22], where the coupling of cloning and sequencing can lead to new deletion mutation in disease, accelerating and reducing costs for patients. Cheaper clone selection methods are needed for applications in diagnostic medicine. 

Expression of proteins still requires tight control, relevant for toxic proteins, which is disadvantageous in induction-free plasmids [23].

Nevertheless, in nature we can find fluorescent proteins that have aided the generation of clone selection plasmids [13] as a good alternative. We found some difficulties with the use of fluorescent reporters: first, colonies can be screened with a source of light in the adequate wavelength that can excite the reporter protein without damaging the DNA inside the cells if the reporter is poorly expressed; second, UV or blue light is needed for the screening of positive clones when colonies are too small and third, the fluorescent signal should be strong (high extinction coefficient) or highly expressed. For these reasons, we explored the possibility of using chromoproteins available in nature. Chromoproteins are GFP-like proteins that have important spectroscopic characteristics (relevant in fluorescent labeling and their applications in imaging) related to their unique spectral, physical, and biochemical properties [24,25]. Studies like the one reported by Alieva and colleagues [24] have uncovered a wide diversity of chromoproteins with a diversity in excitation and emission spectra that can be applied to different reporter applications. We are interested in non-fluorescent purple proteins for two main reasons. First, non-fluorescent purple-blue proteins (called chromoproteins or pocilloporins, proteins that act as photoprotectant in excessive sunlight [25,26], are characterized by high molar extinction coefficient displaying virtually no fluorescence [27], the chromophore possesses another type, which is the isomerized version of the DsRed-like red chromophore [28], where the typical triad Gln-Tyr-Gly is identical to DsRed but the tyrosine ring is non-coplanar and in the *trans* configuration. 

Second, these proteins have been shown to form tetramers [26,28], which render them stable but perhaps not highly useful for protein tagging and subcellular localization as shown for other monomeric fluorescent proteins giving rise to artifacts on protein localization [29].

Third, although fluorescent proteins can also be used as reporter for cloning plasmids when the protein exhibits a broad excitation and emission profile that can be detected by naked eye inspection [30], the confirmation of the phenotype using UV or blue light may be needed with smaller colony forming strains. Also, the simultaneous use of two fluorescent proteins for clone selection and generating a reporter plasmid can be troublesome, therefore, the combination of fluorescent proteins and chromoproteins in cloning plasmids can generate a more versatile system.

With the above information and the need of labs with lower budgets to economize to continue with their research, led us to adapt a marine organism chromoprotein into a cloning system for the following features: (i) the ease to design gene modules, with emphasis on codon usage optimization, transcription and translation elements openly available (http://parts.igem.org/Main_Page); (ii) the reduced costs of DNA synthesis and (iii) the evidence that chromoproteins can be efficiently expressed in *E. coli* (http://parts.igem.org/Main_Page). Here, we report a cloning system that uses the chromoprotein AmilCP as an efficient reporter for cloning plasmids and protein expression plasmids.

## 2. Results

### 2.1. AmilCP as a Reporter for Cloning

**Figure 1:**
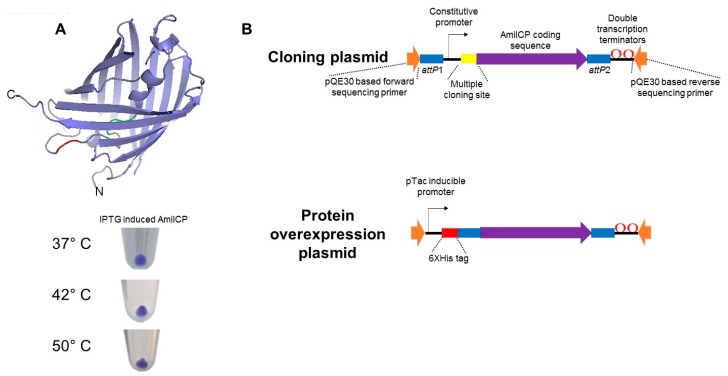
AmilCP predicted structure and plasmid cassette designs. Panel (**A**), predicted structure of AmilCP, the known residues related to GFP fluorescence (Ser65-Tyr70-Pro71) are shown in green, which correspond to residues. The chromoprotein signature of this family of proteins is Gln61, Tyr62 and Gly63, indicated in red [25]. Bellow the predicted structure, cell pellets of *E. coli* expressing cells from an IPTG inducible plasmid (pQE30) incubated at different temperatures for 1 h, showing that the protein retains its color even in stressing temperatures. Panel (**B**), Cassettes designed for cloning and protein expression. All cassettes can be replaced using standard BP Clonase™ reactions.

The use of chromogenic proteins may be limited for protein tagging due to their oligomeric state. AmilCP expressing cells show a strong blue-purple color, which can be readily detected by eye inspection without the need for additional equipment as shown for other systems where high extinction coefficient fluorescent proteins [30]. Sequence analysis showed that this protein is oligomeric, using osFP web server, a specialized software to predict oligomerization state of fluorescent proteins [29]. Also, AmilCP structure is closely related to the GFP family proteins [24] as depicted in Figure 1A, the protein is closely related to the GFP structure showing the predicted β-barrel surrounding the chromophore [24,31]. The residues related to GFP fluorescence (Ser65-Tyr70-Pro71), (green in Figure 1A). Nevertheless, the chromoprotein signature is Gln61, Tyr62 and Gly63 (red in Figure 1A) are present [32]. Also, osFP web server predicts as stated above to form oligomers, most likely tetramers, rendering this protein with limited applications for intracellular localization tagging but may be an excellent reporter protein.

To test the stability of this protein and its toxicity in *E. coli* cells, we expressed AmilCP from a commercial and IPTG inducible plasmid (pQE30, Qiagen, Hilden, Germany). In the cloning process, we used 0.5 mM IPTG in the plates when selecting the transformants. We obtained several blue-purple colonies, showing that the protein when is heavily expressed is non-toxic for *E. coli*. In Figure 1A, we show that cells expressing AmilCP exposed to different incubation temperatures for 1 h retain the blue-purple color, rendering suitable for even high-temperature screening if needed. 

### 2.2. Generation of Cloning Plasmids Using AmilCP Reporter

With the features displayed by AmilCP, we envision to design cloning plasmids that rely on the interruption of the ORF of *amilCP* gene and therefore generate a cloning/screening system independent of both IPTG and a chromogenic substrate such as S-gal or X-gal, thus reducing the expenses in cloning and screening. For such purpose, we designed two plasmid module variants for cloning and one variant for protein expression. In Figure 1B, we show that using intellectual property free parts we were able to assemble three functional genetic modules bearing the necessary elements for expressing AmilCP and cloning. Two cloning plasmids, one containing AmilCP as positive cloning selection marker were created and, to text the efficiency of the *att*P sequences attached to each cassette, another cloning plasmid with JuniperGFP reporter, a protein created by DNA 2.0 and available under the BioBrick Public Agreement (https://www.atum.bio/wp-content/uploads/2013/07/Protein_Paintbox_SynBioPoster_072013.pdf) and intellectual property-free protein openly available (http://parts.igem.org/Part:BBa_J97001), as proof of concept for the use of reporter proteins that can be detected both by naked eye inspection or by UV-light irradiation (Supplementary Figure S1). Using this plasmid, we showed that the Gateway® system is functional using these sequences in our plasmid cassettes (Supplementary Figure S1).

The cassettes were synthesized by ATUM and provided them as cloned fragments, then we removed part of the multiple cloning sites of different plasmids (see Table 1) and replace them with the synthesized cassettes. Upon arrival, all plasmids when transformed provided the resulting colonies with the expected phenotype, colonies showed an intense blue-purple color (AmilCP containing cassettes).

**Figure 2:**
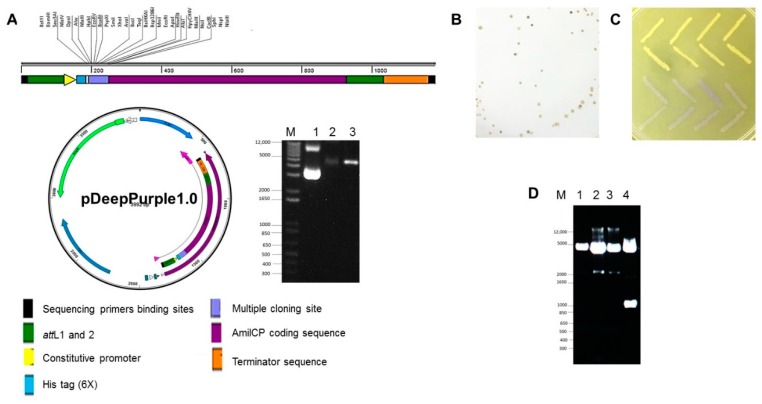
Cloning plasmid pDeepPurple1.0 is functional for cloning applications. Panel (**A**), Plasmid map showing all the restriction sites contained in the multiple cloning site. Also, corroboration of plasmid integrity by restriction analysis using two sites of the multiple cloning site. M, size marker, 1, undigested plasmid; 2, BamHI digested plasmid and 3, XhoI digested plasmid. Panels (**B**,**C**), colonies obtained from the cloning of a 2 and 1 kb fragments from the 1 kb DNA ladder from Invitrogen, cloned into EcoRV restriction site. Panel (**D**), plasmid analysis: lane 1, blue-purple colony, lane 2 and 3, white colonies from the cloning of the 2 kb ladder band and lane 4, white colonies from the cloning of the 1 kb ladder band. M, 1 kb DNA ladder (Invitrogen). The analysis was done using BamHI-HindIII digestion.

The first plasmid we characterized was the pDeepPurple1.0 plasmid designed for cloning applications, bearing the pTZ19R backbone (Fermentas, Burlington, Ontario, Canada) and fully removed the original multiple cloning site (depicted in Figure 2A). The plasmid showed in the agarose gel is undigested and restriction analyzed with two restriction enzymes recognizing two sites in the multiple cloning site showing that the full vector is still under 5 kb, making this plasmid amenable for any cloning applications. In Figure 2B,C we show that the resulting colonies form the ligation with two fragments obtained from the 1kb DNA ladder (Invitrogen) rendered both white and blue-purple colonies, that remained blue-purple once picked and grown again in LB containing only the selection marker for this plasmid (ampicillin). The screening was done on 12 independent colonies of each color and results of three colonies, two for the 2 kb band and one for the 1 kb band is shown in Figure 2D. The success rate was comparable to pTZ19R plasmid standard cloning and screening (data not shown). In the case of pDeepPurple1.0, no false positives were observed, while using pTZ19R when plating 12 colonies that looked white, 2 turned blue and had no insert. The same cassette was cloned into two other vectors, pEXT20 [33] and pGEM T-easy (pDeepPurple1.1 and pDeepPurple1.2 respectively).

**Figure 3:**
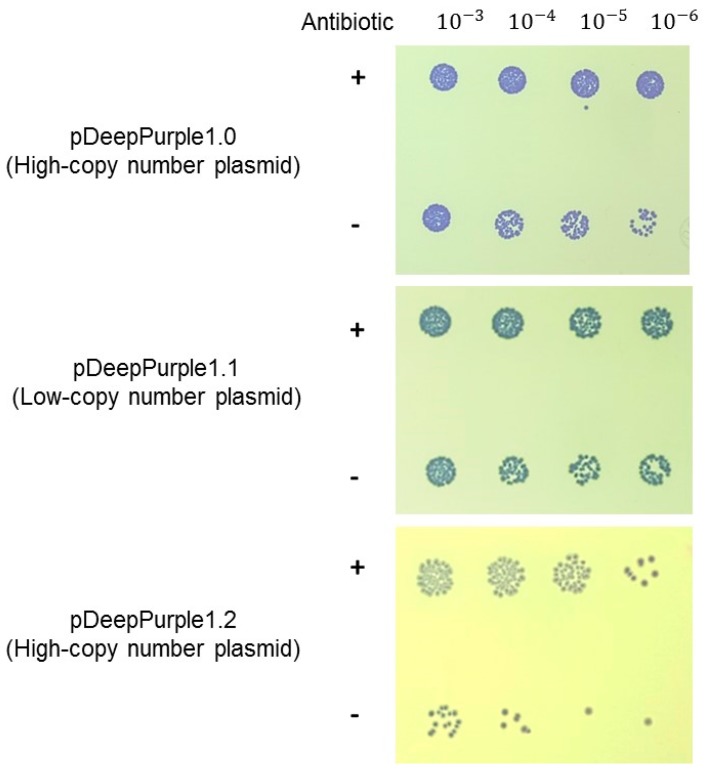
Stability test of the cloning plasmid set. Cells were analyzed as described in Materials and Methods after grown 24 h in the presence (+) or absence (−) of the selection marker (antibiotic). Serial dilutions are indicated on top of the spot analysis. Selection plates contained the selective agent for each plasmid.

Usually, the selection of positive clones using the *lacZ* disruption can render false positives due to some lesser extent mutations (both in the *lacZa* fragment or the chromosomal fragment of *lacZ*) or instability of the plasmid generating a non-functional β-galactosidase enzyme [1,32,34]. We explored the stability of the blue-purple coloration in the cells after containing the pDeepurple1.0 and associated plasmids after 24 h in the absence of selective pressure. In Figure 3, the spot analysis revealed that even in the absence of antibiotic selection, pDeepPurple1.0 and pDeepPurple1.1 showed that most of the cells retained the plasmid when plated again in the presence of the antibiotic. Also, the color of the colonies remained blue-purple, showing a more intense coloration in the high copy number backbone pTZ19R (pDeepPurple1.0). pGEM T-easy backbone seemed to be the lest stable, which may be related to either segregationally loss of the plasmid and more sensitive to be lost during starvation [34] Thus, this system proves to be stable and the remaining cells containing the plasmid expresses correctly AmilCP.

### 2.3. Protein Expression Plasmids Based on AmilCP Cloning Selection

**Figure 4:**
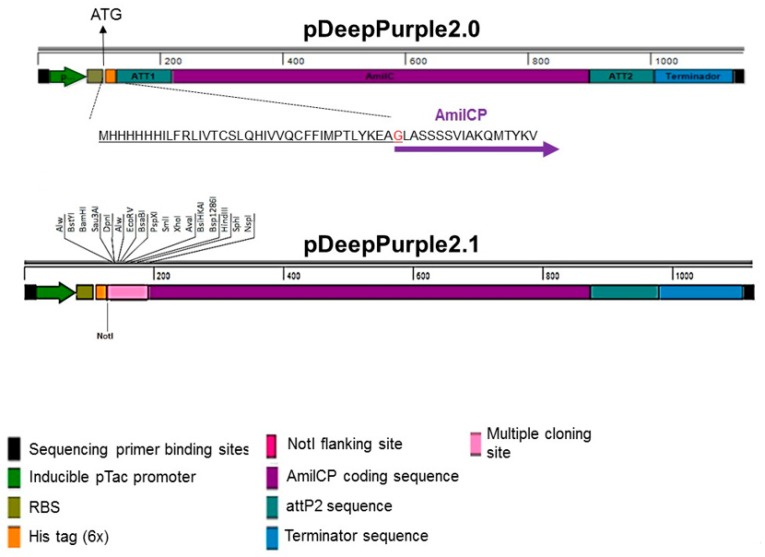
Protein expression cassette map. Original plasmid cassette containing the modified *attP1* for in-frame protein expression. The protein sequence is shown for the modified N-terminal portion of AmilCP.

**Figure 5:**
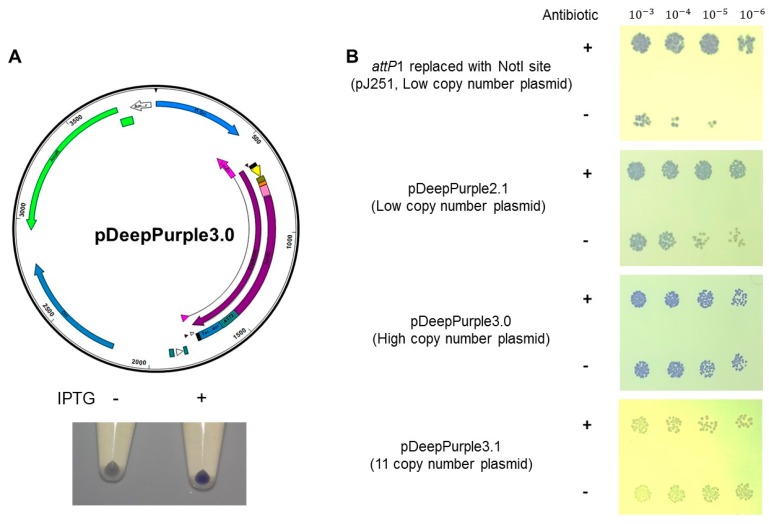
Cloning of the expression cassette to the pTZ19R backbone rendered pDeepPurple3.0. Panel (**A**), plasmid map. Below the map, IPTG induction comparison of AmilCP protein using 0.5 mM in the media for 2 h, (−) indicates cells grown under the same conditions but in the absence of IPTG and (+) indicates induction with IPTG. Panel (**B**) Stability test of the cloning plasmid set. Cells were analyzed as described in Materials and Methods after grown 24 h in the presence (+) or absence (−) of the selection marker (antibiotic). Serial dilutions are indicated on top of the spot analysis. Selection plates contained the selective agent for each plasmid. Here, the intermediate plasmid bearing only the NotI is shown, blue-purple color is present, and no toxicity was observed.

Following the same design, we envisioned for cloning plasmids, we generated a plasmid for protein expression by adding a 6X his tag. We also replaced the constitutive strong promoter used in the previous designs with the hybrid pTac promoter, which is inducible with IPTG [35]. The original design for the protein expression plasmid relied on the use of an in-frame *att*P1 sequence that could express the reporter AmilCP protein and once interrupted, generate white colonies but also maintaining the reading frame for the protein of interest (Figure 4A). Such endeavor required a modified *attP*1 sequence that prevented the ribosome slippage [36]. Upon arrival, we transformed the pJ215 plasmid from ATUM (termed pDeepPurple2.0) containing the designed cassette depicted in Figure 4A, to our surprise, the colonies were all white. We modified the plasmid to generate a functional version that showed intense blue-purple color to achieve clone selection easier. We hypothesized that the *att*P1 sequence affected the oligomeric state of AmilCP, rendering white colonies. Therefore, we replaced the *attP*1 sequence with a NotI restriction site, this modification resulted in blue-purple colonies, even in the presence of IPTG (Figure 4B and Figure 5B). Thus, the absence of color was related to the *attP*1 sequence and not to a mutation associated to AmilCP. This plasmid was further modified by adding an in-frame multiple cloning site (pDeepPurple2.1, Figure 4B) which also resulted in purple colonies. 

With the toxicity problem bypassed, we generated two plasmids, one using the pTZ19R backbone (high copy number plasmid, termed pDeepPurple3.0, Figure 5A) and the pACT3 low copy number plasmid [33] termed pDeepPurple3.1 for the expression of toxic proteins. 

**Figure 6:**
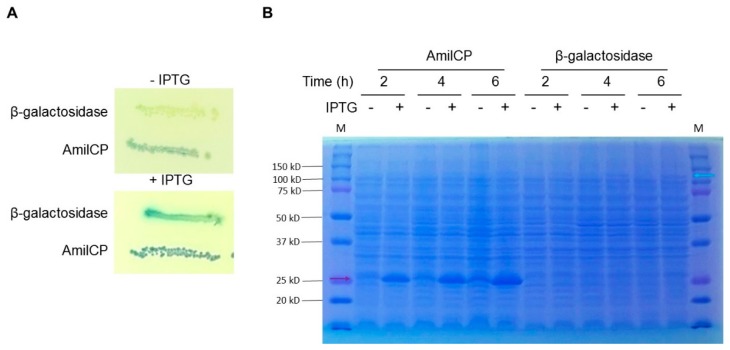
Protein expression is achieved in pDeepPurple3.0 plasmid. Panel (**A**), colony analysis of transformants obtained and the empty vector. One positive colony for β-galactosidase coding sequence was analyzed in the absence (−) or presence (+) of IPTG. In both conditions, plates contained X-gal. Panel (**B**), SDS-PAGE/Coomassie-stained gel electrophoresis analysis of the plasmid expressing only AmilCP (pDeepPurple3.0) or the pDeepPurple3.0 expressing the β-galactosidase enzyme. The induction times are indicated. − and + symbols indicate cells uninduced and induced with IPTG respectively. M, Bio-Rad dual color protein size marker.

In Figure 5A we show the map of the pDeepPurple3.0 plasmid and the response to IPTG for the expression of the reporter AmilCP protein after 2 h induction, observing some leaky expression of the AmilCP reporter. In Figure 5B we show that the resulting plasmids also showed stability and expression of the reporter AmilCP protein in the presence of IPTG after 24 h growth in the absence of the selective agent. In the low-copy-number plasmid pACT3, the color is not so evident by eye inspection. Nevertheless, this low-copy-number plasmid can also be used for toxic protein expression. Our results suggest that the best candidate for overexpressing proteins could be the pDeepPurple3.0 plasmid. To test this, we cloned the encoding sequence for the β-galactosidase enzyme, a 110 kDa protein. We first selected the transformant cells in LB supplemented with ampicillin, X-gal, and IPTG to screen the cells. Then, cells were analyzed in the absence or presence of IPTG. The β-galactosidase activity in the plate was barely detectable in the absence of IPTG, while with the addition of IPTG, the activity was evident (Figure 6A). 

Induction was confirmed by SDS-PAGE, showing that β-galactosidase protein was readily detectable after 2 h induction (Figure 6B), while the control condition, AmilCP is detectable even in the absence of IPTG, suggesting that AmilCP is accumulated in the cell from the leaky expression of the pTac promoter.

### 2.4. Expanding the Application of pDeepPurple1.2 Plasmid

**Figure 7:**
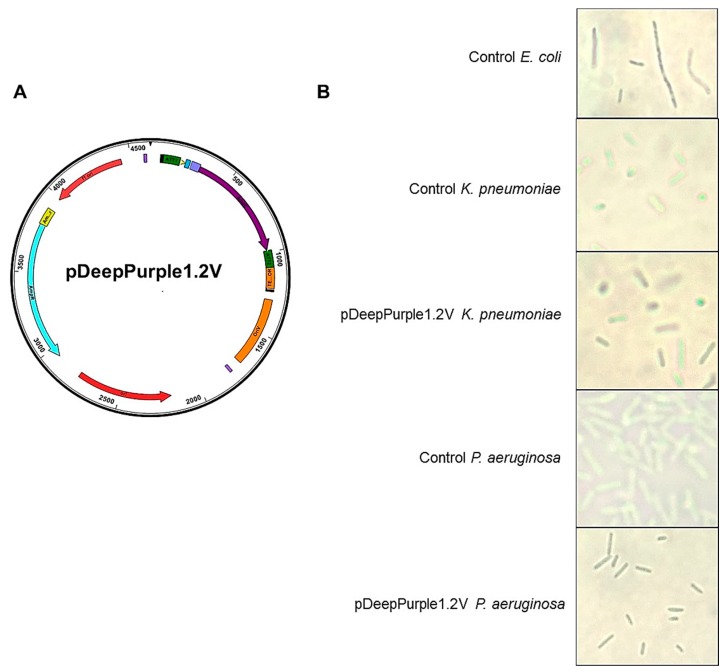
Using AmilCP reporter protein in other bacterial organisms. Panel (**A**)**,** pGEM T-easy vector containing the cloning cassette with AmilCP (pDeepPurple1.2) was modified by incorporating the OriV replication origin. This plasmid was transformed by electroporation into *K. pnreumoniae* and *P. aeruginosa*. Panel (**B**), phenotype analysis by light microscopy (100× magnification) of the transformed strains. Microorganism and plasmid used is indicated. Two different fields are shown, except for the *E. coli* control.

The plasmid pDeepPurple1.2 was further modified by adding the OriV replication origin at SstI restriction site of pGEM T-easy (in close vicinity to the integrated cassette but without disturbing other plasmid elements, Figure 7A). With the resulting plasmid, we transformed *K. pneuomoniae* and *P. aeruginosa* and selected by ampicillin resistance and blue-purple colonies. Two selection passes were done to assess retention of the plasmid. The analysis of the untransformed and transformed strains by light microscopy is shown in Figure 7B. First, the analysis of *E. coli* expressing AmilCP from the pQE30 plasmid showed that the cells under the microscope showed a blue-purple color (Figure 7B). This observation is in concordance with the biochemical analysis currently conducted in our labs, AmilCP has been purified to near homogeneity from the soluble fraction (unpublished data). Second, the cells expressing AmilCP in *K. pneumoniae* or *P. aeruginosa* strains showed a similar color pattern. Third, the strong constitutive promoter used to generate this plasmid is functional in *K. pneumonaie* and *P. aeruginosa* cells. 

With the cumulative data presented here, AmilCP renders an excellent reporter protein for bacterial organisms but requires further research to understand its behavior inside bacterial cells. 

## 3. Discussion

In this work, we generated a set of plasmids designed for cloning and protein expression using a reporter protein that is stable and well expressed in bacteria. Also, the generated plasmid for expressing desired proteins, bearing this reporter protein can facilitate cloning, a feature not commonly found in protein expression vectors. AmilCP protein showed to be a stable and easy to detect. Our results indicate that the cloning plasmids generated using the pTZ19R backbone showed to be the most stable and provided the best results for cloning and positive clone selection. Also, as shown in Figure 7, this system is also applicable to other microorganisms, expanding the molecular tool repertoire for molecular microbiology studies. 

With the design presented here, these plasmids can be used to generate other derivatives for distinct applications by either removing parts of the cassettes or completely remove the reporter coding sequence using the *att* sequences, this feature is true for all the plasmids generated and be further custom modified using the *atPL* sequences flanking the designed cassettes. This is an easy task since the combination of the color phenotype of the transformants, along with the selection markers, make the system flexible. By coupling robotic stations and Gateway^®^ cloning, analysis of several samples can be accelerated and even translated into clinical samples. 

To our knowledge, plasmids relaying on chromoproteins have been reported, using AmilCP in the entry vector, for the Golden-Gate method [37]. Nevertheless, standard cloning that is still used around the globe requires plasmid vectors that are user-friendly with cheaper selection markers. Also, the use of chromogenic markers can lead to novel molecular tools not only for plasmid clone selection but for mutant selection along with other good and reliable positive cloning selection with fluorescent proteins that are readily detected with the naked eye and can be coupled to high throughput strategies [36,37,38]. Taken together, the strategy presented here, which can reduce cloning costs, taking advantage of the also reduced costs for sequencing, can lead to a new cheaper personalized diagnostic medicine [22]. Using the described strategies here, we envision that synthetic biology can benefit all kinds of research and nature has provided all kinds of tools that can be adapted to novel molecular tools.

## 4. Materials and Methods

### 4.1. Cell Culture and Media

Routinely, cells were grown in LB media. Cells transformed with plasmids, LB was supplemented with 50 μg/mL kanamycin for plasmids bearing the pIJ201 vector (ATUM, Table 1) and supplemented with 100 μg/mL ampicillin for plasmids containing either the commercial backbone of pTZ19R or pGEM T-easy or the previously reported pEXT20 and pACT3 expression plasmids [33] (Table 1). When indicated, IPTG was used to a 0.5 mM concentration. Routinely we used XL1-Blue MRF (genotype: Δ((mcrA)183Δ(mcrCB-hsdSMR-mrr)173 recA1 endA1 gyrA96 thi-1 hsdR17 supE44 relA1 lac) strain for cloning and plasmid propagation. *Klebsiella pneumoniae* JH1 strain) and *P. aeruginosa* PA01 strain were grown in LB at 37 °C.

### 4.2. Synthetic Cassettes and Plasmid Constructions

The approach to generate the cloning and expression cassettes consisted in using the standards for cloning BioBricks™ ([39] and http://openwetware.org/wiki/The_BioBricks_Foundation:RFC), but we replaced the need for restriction enzymes due to the 8-bp scar between assembled fragments. To avoid this, we designed the cassettes using iGEM standard parts, eliminated the BioBricks prefix and suffix (in those parts that had them) and assembled them in sillico using NEB Builder^®^ (New England Biolabs, Ipswich, MA, USA) and Gene Designer^®^ (DNA 2.0, now ATUM, Newark, CA, USA) softwares. In Table 2 we provide the complete list of parts used and in Supplementary File 1 the complete sequence file of the plasmids generated in this work. In Figure 1B we depict (not in scale) the cassette modules designed, which were thought to be used in either traditional cloning using restriction enzymes or Gateway^®^ recombination cloning. The coding sequences for AmilCP was codon optimized (Integrated DNA Technologies Codon Optimization tool, http://www.idtdna.com/CodonOpt) for *E. coli* but also with a CAI suitable for other bacterial organisms, assessed with GenScript Rare Codon Analysis Tool (https://www.genscript.com/tools/ rare-codon-analysis). All cassettes were tested first in the ATUM vector (either pIJ201 or pJ215). Then, all cassettes were PCR amplified using the pQE30 sequencing primers and cloned either in pTZ19R, pACT3 or pGEM T-easy plasmids. In the case of pTZ19R, the multiple cloning site was removed as EcoRI-HindIII digestion, filled in and blunt-ended cloned. In the case of pACT3 plasmid, we mutagenized a SspI restriction site contained in the *cat* gene to remove the pTac promoter and multiple cloning site as SspI restriction and blunt-ended cloning of the desired cassette. Finally, pGEM T-easy was digested with SphI and NdeI, blunt ended with Klenow fragment and then cloned also as blunt-ended the desired cassette. For further characterization of AmilCP, we generated an independent construct by cloning the coding sequence of AmilCP into the pQE30 plasmid (Qiagen) as a BamHI-HindIII fragment. In Table 3 we listed the primers used in this study. All PCR reactions were conducted using Herculase II fusion DNA polymerase (Agilent Technologies, USA, Ceedar Creek, TX, USA) using the standard amplification protocol: 94 °C for 2 min, 30 cycles of 94 °C for 20 s, 50 °C for 30 s and 72 °C for 1 min due to the sizes of the cassettes used in this study. 

The modified plasmid for other hosts, besides *E. coli*, was created by PCR amplifying the OriV [40] from a synthesized gBlock based on the BBa_K125340 sequence. SstI pTZ19R bearing the AmilCP cloning cassette (named pDeppPurple1.0) was blunt ended with Klenow and ligated to the blunt-ended OriV PCR fragment. Transformants were selected in *E. coli* and PCR verified.

### 4.3. Chromogenic Plasmid Stability Analysis

To determine qualitatively the number of remaining cells carrying the plasmids generated after selective agent removal for 24 h, we conducted a spot test. First, cells were grown in the presence of the selective agent overnight. Second, cells were diluted to an OD_600_ of 0.2 in LB without a selective agent and incubated for 24 h. The control condition was done equally but in the presence of the selective agent. Finally, cells were serial diluted as indicated in the figures in LB medium. Three microliters of these cell suspensions were spotted on LB agar plates and incubated for 16 h at 37 °C, plate image was recorded using an Image Station 2000R (KODAK, Rochester, NY, USA). Experiments were performed in triplicate.

### 4.4. Cloning Test

To demonstrate that the cloning system is effective, the pDeepPurple1 plasmid was digested with EcoRV. 1 kb ladder (Invitrogen) size marker bands (2 and 1 kb) were gel purified and blunt-ended with Klenow DNA polymerase. The fragments were ligated and then transformed. Screening of colonies was performed by blue-purple/white discrimination. Standard miniprep plasmid purification was carried and BamHI-HindIII restriction analysis was done to confirm the presences of either the 2 kb or 1 kb band.

### 4.5. Protein Expression Test in pDeepPurple 3.0

To test protein overexpression, we chose the β-galactosidase enzyme as proof of concept. This is a 110 kDa protein that we can also trace for activity. We cloned as a BamHI and HindIII fragment the whole coding sequence into pDeepPurple3.0 plasmid and screened the transformants with X-gal and IPTG containing plates. Expression was tested in LB with 0.5 mM IPTG for 2, 4 and 6 h. Cells were collected by centrifugation by taking 1 mL of cell culture, resuspended in PBS 1X. Cells were lysed in 2X loading buffer, boiled for 5 min. and loaded into a 12% SDS page to compare both AmilCP (25.4 kDa) and β-galactosidase (110 kDa) enzyme.

### 4.6. Plasmid Mutagenesis

The protein expression cassette was mutagenized using the QuikChange II Site-Directed Mutagenesis Kit (Agilent) following the manufacturer’s instructions and using the primers listed in Table 3. First, the protein expression cassette was mutagenized to eliminate *attP*1 sequences and replacing it with a NotI restriction site. Then, the multiple cloning site was added by aligning the primers listed in Table 3 MCS1 and MCS2 that once aligned left 5’ and 3’ ends compatible to NotI and regenerated the same restriction site. The pACT3 was mutated to remove the SspI restriction site at the *cat* gene using the same strategy and primers mutsspIpact3 and mutsspIpact3compl (Table 3).

### 4.7. Protein Sequence and Structural Analysis

AmilCP protein sequence was analyzed using osFP web server for oligomerization prediction [30]. Protein structure was modeled using the Phyre2 web server. Protein surface was estimated using DeepView-Swiss PDB viewer [41] to calculate distances and hydrophobicity. Also, using Phyre2 webserver we estimated the conservation of protein models [42,43], visualization was done with PyMol software version 1.8 [44].

### 4.8. K. preumoniae and P. aeruginosa Transformation

Both strains were tested for ampicillin resistance. *K. pneuomniae* JH1 strain showed sensitivity to 100 μg/ml, while the *P. aeruginosa* PA01 strain showed sensitivity at 300 μg/mL. Cells were electroporated according to the protocols of the Gene Pulser Xcell™ electroporation system (Bio-Rad, USA, Hercules, CA, USA) without any modification. Electroporation was carefully done on each strain using 2 μg of purified plasmid pDeppPurple1.2V, bearing the OriV replication origin. pGEM’T-easy backbone was the only vector in our set that permitted us to do this cloning. Transformed cells were selected in LB media with the corresponding amount of ampicillin on each plate. Two replica plating was used to isolate blue-purple colonies and analyzed by light microscopy (Axioskop 40, ZEISS, USA, Thornwood, NY, USA) using 100× amplification with immersion oil.

## Figures and Tables

**Table 1:** Plasmids generated in this study.

Plasmid name	Backbone	Features	Antibiotic Selection Marker
pDeepPurple1.0	pTZ19R	High copy number cloning plasmid	Ampicillin
pDeepPurple1.1	pEXT20	40 copy cloning plasmid	Ampicillin
pDeepPurple1.2	pGEM T-easy	High copy number cloning plasmid	Ampicillin
pDeepPurple1.2V	pGEM T-easy	High copy number cloning plasmid, *ori*V sequence present rendering a plasmid selectable in other bacteria besides *E. coli*	Ampicillin
pJ251 *attP*1 replaced with a NotI restriction site	pJ251	low copy number cloning plasmid modified from the original design for protein expression, only NotI restriction site present for cloning desired gene	Kanamycin
pDeepPurple2.1	pJ251	Low copy number cloning plasmid for protein expression, multiple cloning site present	Kanamycin
pDeepPurple3.0	pTZ19R	High copy number cloning plasmid for protein expression, multiple cloning site present	Ampicillin
pDeepPurple3.1	pACT3	11 copy cloning plasmid for protein expression, multiple cloning site present	Chloramphenicol

**Table 2:** Parts used to generate the plasmids in this study.

Part Name	Accession Number or Identification Number	Features	Purpose
Promoter sequence (Constitutive)	BBa_J23100	35 bp strong constitutive promoter	Constitutive expression of AmilCP protein for clone selection (Blue/White screening).
Promoter sequence (*tac* inducible promoter, functional hybrid derived from the *trp* and *lac* promoters)	BBa_K864400	61 bp inducible promoter	Inducible expression of proteins in expression plasmids [35].
RBS	BBa_B0035	27 bp ribosome binding site, optimal for prokaryotic organisms	Constitutive expression of AmilCP protein for clone selection (Blue/White screening).
Terminator sequence	BBa_B0015	129 bp terminator sequence	Constitutive expression of AmilCP protein for clone selection (Blue/White screening) and protein expression plasmids.
*att* sequences	Invitrogen Gateway^®^	*att* sequences for recombination cloning (Gateway® system)	Cloning in vectors containing *att* sequences. Although, expression plasmid is limited due to toxicity generated by *attL2* sequence (see text).
OriV	BBa_K125340	424 bp fragment, origin of replication. Broad host capabilities	Generate a plasmid capable of replicating in other hosts besides *E. coli*.

**Table 3:** Oligonucleotides used in this study.

Primer Name	5’ to 3’ Sequence	Application
F forward	CCCCGAAAAGTGCCACCT	Primer pair used to amplify all the designed cassettes and cloning them into different plasmid backbones
F reverse	GTTCTGAGGTCATTACTGGTATAAACG	
OriV Fwd	AACCCCTGCAATAACTGTC	Primer pair used to amplify the *ori*V replication origin from a gBlock synthesized fragment
OriV Rv	GCTGAATGATCGACCGAG	
NotIinsertionFw	ACTAGATGCATCACCATCACCATCACGCG GCCGCTGGCTTCCAGCAGTTCGGTGATTGCG	Primer pair to replace the *attP*1 sequence in the protein expression cassette and replace it with a multiple cloning site
NotIinsertionRv	CGCAATCACCGAACTGCTGGAAGCCAGCGG CCGCGTGATGGTGATGGTGATGCATCTAGT	
MCS1	GGCCGCGGATCCCATGATATCCATCACCTCGA GCACCACCACAAGCTTCATGCATGCGC	Primer pair to generate a multiple cloning site for the protein expression cassette. Once aligned, the ends are compatible to a NotI digested plasmid.
MCS2	CGCCGGGCATGCATGAAGCTTGTGGTGGTG CTCGAGGTGATGGATATCATGGGATCCGC	
MutsspIpact3	CCTTGCGTATAAAATTTGCCCATGGTG	Primer pair to eliminate SspI restriction site from the *cat* gene in pACT3 plasmid.
MutsspIpact3compl	CACCATGGGCAAATTTTATACGCAAGG	
CaroFw	GGGGCCAACTTTGTACAAAGAAGCA GGCTATGCCCTTTACCATTGATAG	Primer pair to amplify and clone the water-soluble carotenoid protein sequence using the Gateway^®^ cloning system.
CaroRv	GGGGACCACTTTGTACAAGAAAGCTG GGTCTAACGGGCAAAATTCAAAAG	

## Supplementary Materials

The following are available online. Figure S1: Using protein with color and fluorescence for cloning. In supplementary text file we provide the information for the JuniperGFP-based Gateway^®^ cloning plasmid and the full sequence of the most used plasmids in this study. In supplementary Figure S1 we provide the analysis of a JuniperGFP-based Gateway^®^ cloning plasmid.
